# Twelve-Month Follow-Up to a Fully Automated Internet-Based Cognitive Behavior Therapy Intervention for Rural Adults With Depression Symptoms: Single-Arm Longitudinal Study

**DOI:** 10.2196/21336

**Published:** 2020-10-02

**Authors:** Mark Schure, Bernadette McCrory, Kathryn Tuchscherer Franklin, John Greist, Ruth Striegel Weissman

**Affiliations:** 1 Department of Health & Human Development Montana State University Bozeman, MT United States; 2 Department of Mechanical & Industrial Engineering Montana State University Bozeman, MT United States; 3 Center for Mental Health Research and Recovery Montana State University Bozeman, MT United States

**Keywords:** internet-based cognitive behavior therapy, depression, anxiety, long-term outcomes, iCBT, CBT, therapy, mental health, outcome

## Abstract

**Background:**

Internet-based cognitive behavior therapy (iCBT) interventions have the potential to help individuals with depression, regardless of time and location. Yet, limited information exists on the longer-term (>6 months) effects of iCBT and adherence to these interventions.

**Objective:**

The primary aim of this study was to evaluate the longitudinal (12 months) effectiveness of a fully automated, self-guided iCBT intervention called Thrive, designed to enhance engagement with a rural population of adults with depression symptoms. The secondary aim was to determine whether the program adherence enhanced the effectiveness of the Thrive intervention.

**Methods:**

We analyzed data from 181 adults who used the Thrive intervention. Using self-reports, participants were evaluated at baseline, 8 weeks, 6 months, and 12 months for the primary outcome of depression symptom severity using the Patient Health Questionnaire-9 (PHQ-9) scale and secondary outcome measures, namely, the Generalized Anxiety Disorder Scale-7 (GAD-7) scores, Work and Social Adjustment Scale (WSAS) scores, Conner-Davidson Resilience Scale-10 (CD-RISC-10) scores, and suicidal ideation (ninth item of the PHQ-9 scale) scores. The Thrive program adherence was measured using the numbers of program logins, page views, and lessons completed.

**Results:**

The assessment response rates for 8-week, 6-month, and 12-month outcomes were 58.6% (106/181), 50.3% (91/181), and 51.4% (93/181), respectively. By 8 weeks, significant improvements were observed for all outcome measures. These improvements were maintained at 12 months with mean reductions in severities of depression (mean –6.5; *P*<.001) and anxiety symptoms (mean –4.3; *P*<.001). Improvements were also observed in work and social functioning (mean –6.9; *P*<.001) and resilience (mean 4.3; *P*<.001). Marked decreases were observed in suicidal ideation (PHQ-9 ninth item score >1) at 6 months (16.5%) and 12 months (17.2%) compared to baseline (39.8%) (*P*<.001). In regard to the program adherence, cumulative counts of page views and lessons completed were significantly related to lower PHQ-9, GAD-7, and WSAS scores and higher CD-RISC-10 scores (all *P* values <.001 with an exception of page views with WSAS for which *P* value was .02).

**Conclusions:**

The Thrive intervention was effective at reducing depression and anxiety symptom severity and improving functioning and resilience among a population of adults from mostly rural communities in the United States. These gains were maintained at 1 year. Program adherence, measured by the number of logins and lessons completed, indicates that users who engage more with the program benefit more from the intervention.

**Trial Registration:**

ClinicalTrials.gov NCT03244878; https://clinicaltrials.gov/ct2/show/NCT03244878

## Introduction

Clinician-delivered cognitive behavior therapy (CBT) is a long-standing evidence-based psychotherapy for depression and anxiety symptoms and disorders [1,2]. Computerized forms of CBT were introduced three decades ago, paving the way for present efforts to implement such programs via the internet [3]. Compared to clinician-delivered CBT, internet-based cognitive behavior therapy (iCBT) programs have potential for greater reach and scalability, greater standardization of content delivery, and reduced risk of stigmatization [4-6]. Even more, they have demonstrated equivalent effectiveness for reducing depression and anxiety symptoms [7].

Studies support the feasibility, acceptability, and effectiveness of self-guided (no supportive contacts by email, text, telephone, or face-to-face) iCBT interventions on depression and anxiety symptoms [4,6,8-10]. These findings are particularly promising for people living in rural and frontier communities, which, nationally and internationally, have greater behavioral health care access challenges [11-13]. Compared with urban residents, rural and frontier residents have fewer qualified mental and behavioral health care providers and longer travel times to clinical services; moreover, they report greater concerns about privacy and higher levels of stigma [8,14-16]. Outside of the United States, other identified barriers to care in rural settings include long wait times for appointments, cost of care, transportation, lack of education, and stigma toward seeking mental health care [17,18].

A meta-analysis of 13 randomized controlled trials (RCTs), all conducted outside the United States [4], evaluated the efficacy of self-guided iCBT interventions for adults with depression symptoms. Compared to controls, iCBT was significantly more effective for the intervention groups on depressive symptoms severity. Furthermore, program adherence was significantly associated with reduction in depression symptoms. Another meta-analysis of 64 RCTs evaluated the effectiveness of iCBT, usually delivered with support from a health care provider or other individual, compared to the usual care, wait list, or placebo intervention in the treatment of depression or anxiety [6]. The meta-analysis reported superior outcomes among the intervention group compared to the control group. Most self-guided iCBT trials with outcomes of depression and anxiety have shorter follow-up assessment periods (≤6 months). We identified only 2 studies reporting longer-term effects of completely self-guided iCBT on depression symptoms. In Mira and colleagues’ trial [19], 12-month effect sizes for depression symptoms demonstrated a moderate within-group effect size (*d*= 0.67). Clarke and colleagues [20] reported on an 8-month follow-up on depression symptoms, indicating a comparable effect size of 0.72 (calculated by authors).

In the context of online programming for mental health interventions, adherence has been defined as “the extent to which individuals experience or engage with content” [21]. However, operationalizing adherence to iCBT interventions poses challenges. For example, while in-person CBT typically involves treatment sessions (with a mutually agreed upon start and end time), electronic delivery of treatment has varying degrees of engagement and length of use. The content of iCBT may be designed to comprise numerous short videos that can be viewed in a variety of temporal orders and explored in a variety of timeframes different from what would typically be encountered in an in-person session. Hence, the concept of a session may not easily translate from in-person CBT to iCBT or from one iCBT program to another.

Most published RCTs evaluating the efficacy of iCBT interventions have been implemented in non-US urban settings. Our research team evaluated an iCBT intervention, called Thrive, designed to help improve depression and anxiety symptoms for adults residing in the western rural communities in the United States [8,9]. Thrive is an interactive, fully automated, self-guided intervention that uses a video-based platform to deliver CBT curriculum and provide supportive feedback to users [22]. We implemented a pragmatic usual care waitlist control (WLC) RCT, enrolling 343 adults with at least mild depression symptoms (a Patient Health Questionnaire-9 [PHQ-9] score >5), who were randomized either to immediate access to the Thrive intervention or to 8-week delayed access [9]. In models adjusted for potential confounders, depression severity following 8 weeks of intervention was significantly lower for the immediate access group than for the WLC group (*d*= 0.63). Superior 8-week outcomes in the immediate access group versus WLC group were also observed for secondary outcomes, including anxiety symptoms (*d*=0.47), work and social functioning (*d*=0.39), and resilience (*d*=0.55). Although not statistically significant, the immediate access group was 45% less likely (odds ratio [OR] 0.55) to report suicidal ideation compared to the WLC group.

Since there are few studies examining the long-term impact of self-guided iCBT interventions, the primary aim of this within-group analysis was to assess 6- and 12-month follow-up outcomes of trial participants who received immediate access to the Thrive intervention. Moreover, to date, there has been no consensus on the operationalization or impact of adherence on outcomes in iCBT interventions [21]. Thus, our secondary aim was to determine whether program adherence enhanced the effectiveness of the Thrive intervention.

## Methods

### Trial Design

A longitudinal study design was used to evaluate the effectiveness of the Thrive intervention with participants receiving immediate access to the iCBT program. Study participants were enrolled in the study between September 2017 and January 2018 and were provided free online access to the intervention for 1 year. The original study design was a waitlist RCT in which participants were randomly assigned to receive either an immediate access to the iCBT intervention or access to the intervention delayed 8 weeks. The WLC group received a link to the National Institute of Mental Health’s depression information webpage. All participants received a link to the resource webpage of Montana’s chapter of the National Alliance on Mental Illness. All participants were also permitted to continue or begin whatever usual care was available to them. The Montana State University Institutional Review Board (IRB) approved the protocol and all related materials (#MS033017-FC) prior to study initiation. The study is registered at ClinicalTrials.gov (NCT03244878).

### Study Recruitment

We used several strategies for promoting the study to state residents. We first partnered with Montana State University Extension faculty to disseminate study brochures and flyers that guided potential participants to the study website, where they could learn more and sign up. Research team members also conducted 12 community meetings throughout all regions of the state, which were promoted by local extension agents. Other recruitment sources included public service announcements, local newspaper articles, social media (Facebook and Craigslist community pages), email listservs, large employers, and local health care providers.

### Study Eligibility and Participants

Eligibility requirements for the study included age >18 years; Montana state residency; having regular access to broadband internet via a computer, tablet, or smartphone; and reporting at least mild depression symptoms (PHQ-9 score >5) at baseline. Potential participants were directed to a study website where they were informed about study participation; self-screened for eligibility; and, if eligible, guided through the informed consent, randomization, and online assessment process [23]. The Montana State University logo was displayed throughout the study website pages. Of the 573 individuals assessed for eligibility, 463 individuals were deemed study eligible and enrolled in the study; yet 109 were later identified as fraudulent and removed from the study. The sample for the current study included 181 eligible study participants who had immediate access to the Thrive intervention (see Multimedia Appendix 1 for the CONSORT (Consolidated Standards of Reporting Trials) flowchart).

### Intervention Description

Thrive, developed by Waypoint Health Innovations [22], is a self-guided iCBT intervention for depression and anxiety that distills best practices from CBT and delivers them through a rich, structured, and guided curriculum. Thrive uses video, interactive tools, and sophisticated algorithms that dynamically adjust the individual’s course through the intervention. The intervention is comprised of 320 videos, averaging 80 seconds in length, to deliver content. Videos explain CBT concepts, demonstrate skills, provide feedback and recommendations, and portray actual case histories of individuals who used CBT skills to improve depression symptoms. The intervention also provides periodic PHQ-9 self-assessments and tailored feedback based on the scores. For this study, over a third of the demonstration and case history videos were replaced with new videos featuring rural characters, story lines, and settings. Other features of the Thrive program (ie, didactic and feedback videos, interactive tools, and algorithms) were not modified for this study. Thrive incorporates classic cognitive behavior therapy themes in modules (series of the didactic and feedback videos and interactive tools) on Constructive Thinking (cognitive restructuring), Pleasant Activities (behavioral activation), and Assertive Communication (social skills training). Each module has 10 lessons and suggested exercises for users to practice offline as homework pertinent to their own goals.

The cost for an individual to use Thrive for 6 months is roughly equivalent to the cost of 1 session with a therapist.

### Assessments

All participants were assessed at baseline, 8 weeks, 6 months, and 12 months after study enrollment for each outcome measure. Each participant received email reminders when assessments were due, and 2 additional reminders within 7 days were issued for those who had not yet completed their assessment. Data were designated as lost to follow-up when no assessment was completed within 10 days of the due date. Participants with completed interim assessments were rewarded with a US $25 Amazon gift code, and those with the completed final assessment (12 months) were rewarded with a US $30 Amazon gift code.

### Measures

All outcome measures and other demographic and treatment measures were administered electronically via the study assessment portal. The primary outcome measure was depression symptom severity measured by the PHQ-9, (score range 0-27; higher scores indicate greater severity) [24]. Secondary outcome measures included anxiety symptom severity, daily functioning, resilience, and suicidal ideation. Anxiety symptom severity was measured with the Generalized Anxiety Disorder Scale-7 (GAD-7) (score range 0-21; higher scores indicate greater severity) [25]. Daily functioning was measured with the Work and Social Adjustment Scale (WSAS) (score range 0-40; higher scores indicate worse daily functioning) [26]. Resilience was measured with the Connor-Davidson Resilience Scale-10 (CD-RISC-10) (score range 0-40; higher scores indicate greater resilience) [27]. Frequency of suicidal ideation was measured with the ninth item of the PHQ-9 (score range 0-3; higher scores indicate greater suicidal ideation).

We assessed rates of remission and relapse. Remission was defined as a treatment response in which an individual with mild, moderate, moderately severe, or severe depression at baseline (PHQ-9 scores ≥5) achieved a subsequent PHQ-9 score lower than 5 at 6 months and 12 months. Relapse was defined as a PHQ-9 score ≥10 for those who had achieved remission at 6 months or 12 months. Cumulative program adherence was measured with the following indicators: (1) number of logins, (2) number of page views, and (3) number of lessons completed within the program. Cumulative counts of each of these 3 program usage measures were assessed as explanatory variables for each of the 4 continuous outcome measures (PHQ-9, GAD-7, WSAS, CD-RISC-10) and the PHQ-9 ninth item, suicidal ideation.

Demographic variables included age (years), gender (female vs male), race (White vs other), marital status (single vs married/domestic relationship), employment status (employed full-time, employed part-time, unemployed/retired/student), veteran status (yes vs no), educational attainment (≤ college without a degree, bachelor’s degree, ≥ master’s degree), health insurance (private, public, other, none), and rural classification (urban, large rural, small rural, isolated).

### Participant Safety

Participants were encouraged to seek or continue other available care throughout the study. During all assessments, participants who reported any frequency of suicidal ideation (PHQ-9 ninth item score >0) on the assessment portal were encouraged to seek help from multiple sources. They were also asked whether they could keep themselves safe from self-harm. Those responding they could not keep themselves safe would be told not to continue in the study and were provided a list of things to do to seek professional help. All participants were provided a resource list for seeking additional support (Multimedia Appendix 2). However, none of the participants responded they could not be safe during any assessment. Additionally, in the Thrive intervention, any self-assessed PHQ-9 scores >20 and a PHQ-9 score >10 on the third self-assessment recommended seeking a clinician’s help. Participants were also provided contact information of the institution’s IRB director and the study’s principal investigator to report any adverse events. No adverse events were reported in this study.

### Statistical Analysis

The longitudinal change over time in each continuous outcome was assessed using a linear mixed model analysis of repeated measures. Separate models were created for each outcome measure (ie, PHQ-9, GAD-7, WSAS, and CD-RISC-10) to assess the fixed effect of time adjusting for baseline scores and receiving therapy for depression. Similar separate models were used to assess the relationship program logins, page views, and lessons completed on each outcome, adjusting for baseline scores, therapy for depression, and time.

The PHQ-9 ninth item, suicidal ideation, was treated as an ordinal outcome that ranged from 0 (“not at all,” no suicidal ideation) to 3 (“nearly every day”) where the cumulative probabilities were modeled over the higher-ordered suicidal ideation scale scores (indicating greater suicidal ideation). Using an ordinal logistic regression model within a generalized estimating equation framework, the PHQ-9 ninth item was assessed with the fixed effect of time adjusting for baseline scores and receiving therapy for depression. Similar separate ordinal logistic regression models were used to assess the relationship program logins, page views, and lessons completed, adjusting for baseline scores, therapy for depression, and time.

Statistical analyses were performed using SAS software, version 9.4 (SAS/STAT 14.2, SAS Institute Inc). Maximum likelihood estimators allow efficient parameter estimation using only available data under an assumption of missing at random [28-30]. Sensitivity analyses were also conducted using only participants with complete data (ie, complete cases), and no significant differences were found between those who were lost to follow-up/noncompleters and those who completed the trial. Attrition/loss to follow-up was assessed to ensure the key covariates, and baseline measures did not differ from those that completed and those that did not complete the trial. The baseline primary and secondary outcomes each showed no significant differences between trial completers and noncompleters. There were no significant differences found among completers for each outcome variable of interest based on age, gender, marital status, employment, rural-urban commuting area codes, or baseline therapy usage. Due to extremely low sample sizes, variables of race, veteran status, and other or no insurance could not be assessed. The level of significance was set at α=.05 (two-tailed), and the Bonferroni method was implemented to control false positives over the multiple tests.

## Results

### Participant Characteristics

A total of 181 immediate intervention group participants (iCBT) were included in this longitudinal outcome assessment. As detailed in Table 1, participants were on average 42 years old (SD 12.8); and most were female (88.9%), White (93.9%), and nonveterans (96.1%). A majority was married or in a domestic relationship (56.9%), employed full-time (61.9%), had obtained at least a bachelor’s degree (56.9%), and had private health insurance (77.3%). Nearly 15% of participants lived in urban, over 56% in rural, and nearly 29% in isolated communities. Nearly 57% of participants reported receiving clinical care for mental health.

**Table 1:** Baseline characteristics of the analytic sample (N=181).

Characteristics		Values
Age (years), mean (SD)		42.1 (12.8)
Female, n (%)		160 (88.4)
**Race, n (%)**		
	White	170 (93.9)
	Other	11 (6.1)
**Marital status, n (%)**		
	Single	78 (43.1)
	Married/domestic partnership	103 (56.9)
**Employment status, n (%)**		
	Employed full-time	112 (61.9)
	Employed part-time	39 (21.6)
	Unemployed/retired/student	30 (16.8)
Veteran, n (%)		7 (3.9)
**Education, n (%)**		
	Some college or less	78 (43.1)
	Bachelor's degree	61 (33.7)
	Graduate or professional degree	42 (23.2)
**Health insurance, n (%)**		
	Private	140 (77.3)
	Public	32 (17.7)
	Other	5 (2.8)
	None	4 (2.2)
**Rural classification^a^, n (%)**		
	Urban	27 (14.9)
	Large rural	42 (23.2)
	Small rural	60 (33.2)
	Isolated	52 (28.7)
Receiving mental health treatment^b^, n (%)		103 (56.9)

^a^Defined using the rural-urban commuting area codes.

^b^Defined as receiving any clinical care or taking medication(s) for depression symptoms.

### Clinical Outcomes

The assessment response rates for the 8-week, 6-month, and 12-month outcomes were 58.6%, 50.3%, and 51.4%, respectively. Thus, the respective attrition rates were 41.4%, 49.7%, and 48.6%. To assess remission and relapse, participants’ PHQ-9 scores were assessed for changes from baseline to 8 weeks, 6 months, and 12 months. Of the 107 participants who completed week 8 assessments, 42 (39.3%) achieved remission. Among the 42 participants who achieved remission, 22 (52.4%) and 24 (57.1%) maintained remission at 6 months and 12 months, respectively. Only 4 participants (9.5%) who had achieved remission at week 8 subsequently relapsed at 6 months (n=2) and 12 months (n=2).

Further, 72.9% (78/107) participants had moderate or greater depression symptoms at baseline (PHQ-9 score ≥10). From this subgroup of 78, 28 (35.9%) achieved remission at week 8. Of these 28 participants who achieved remission within this subgroup, 11 (39.3%) and 13 (46.4%) maintained remission at 6 months and 12 months, respectively. Only 4 (14.2%) participants who had achieved remission at week 8 subsequently relapsed at 6 months (n=2) and 12 months (n=2).

Longitudinal mean outcome scores and adherence metrics are presented in Table 2.

**Table 2:** Longitudinal mean trend of clinical and adherence measures.

Measures			Baseline, mean (SD)		8 weeks, mean (SD)		6 months, mean (SD)		12 months, mean (SD)		Significance^a^		
										*F* test (*df*)		*P* value (adj)	
Primary outcome measure (Depression symptom severity)^b^			13.7 (5.0)		7.2 (5.1)		7.0 (5.4)		7.2 (5.6)		121.6 (3)		<.001
**Secondary outcome measures**													
	Anxiety symptom severity^c^	10.3 (4.7)		6.4 (4.7)		6.2 (4.8)		6.0 (5.4)		50.4 (3)		<.001	
	Work and social functioning^d^	20.2 (8.0)		15.2 (9.4)		13.7 (9.7)		13.3 (10.5)		29.1 (3)		<.001	
	Resilience^e^	22.2 (6.6)		25.6 6.1)		26.1 (6.3)		26.5 (6.7)		28.9 (3)		<.001	
	Suicidal ideation^f^ (score ≥1), n (%)	72 (39.8)		13 (12.2)		15 (16.5)		16 (17.2)		48.9 (3)^g^		<.001^h^	
**Adherence measures**													
	Logins (cumulative counts)	N/A^i^		8.9 (9.6)		11.1 (13.3)		11.7 (14.3)		N/A		N/A	
	Page views (cumulative counts)	N/A		66.3 (70.4)		80.9 (89.1)		84.8 (93.5)		N/A		N/A	
	Lessons completed (cumulative counts)	N/A		7.7 (7.9)		8.9 (9.1)		9.2 (9.3)		N/A		N/A	

^a^*P* value was associated with the test (*F* test, Type 3 tests of fixed effects) of the overall time period (week) difference. *P* values adjusted by the Bonferroni method (adj) are shown.

^b^Patient Health Questionnaire-9 (PHQ-9) score range = 0 to 27.

^c^Generalized Anxiety Disorder Scale-7 (GAD-7) score range = 0 to 21.

^d^Work and Social Adjustment Scale (WSAS) score range = 0 to 40.

^e^Connor-Davidson Resilience Scale-10 (CD-RISC-10) score range = 0 to 40.

^f^PHQ-9 Item 9, Suicidal Ideation, score range = 0 to 3.

^g^Chi-square statistic, χ^2^ (degrees of freedom).

^h^*P* value was associated with the test (χ^2^, Type 3 generalized estimating equations analysis) of the overall time period (week) difference. *P* value adjusted by the Bonferroni method (adj) is shown.

^i^N/A: not applicable.

**Figure 1:**
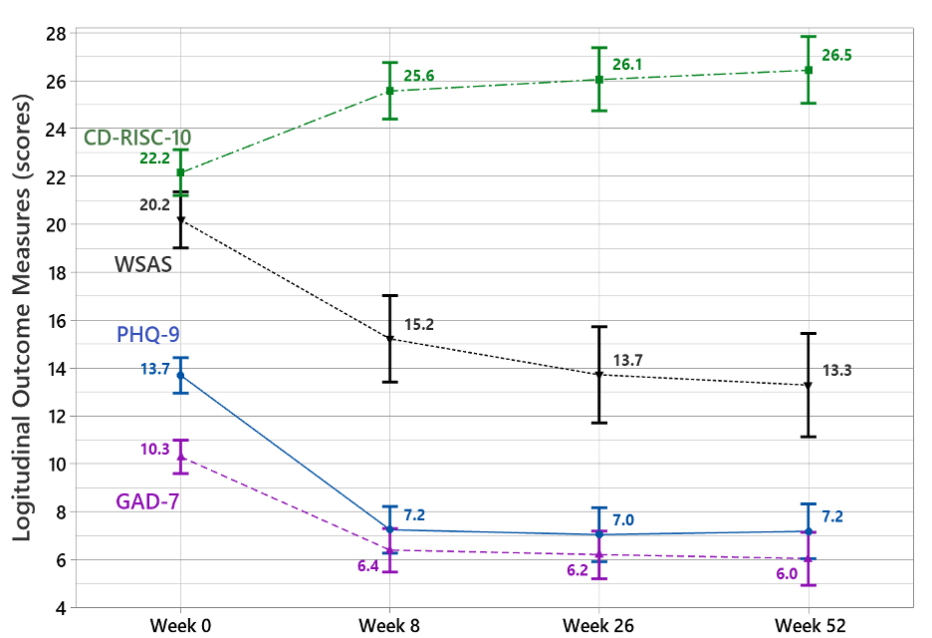
Mean effect of treatment over time by clinical measure with 95% CI. CD-RISC-10: Conner-Davidson Resilience Scale-10; GAD-7: Generalized Anxiety Disorder scale-7; PHQ-9: Patient Health Questionnaire-9; WSAS: Work and Social Adjustment Scale.

Mean effect of treatment over time by outcome measure with 95% CI is illustrated in Figure 1.

By 8 weeks, significant improvements were observed for all outcome measures. These improvements were maintained at 6 and 12 months. We observed 6-month mean reductions and respective effect sizes in the severity of depression (mean –6.3; *d*=1.27) and anxiety symptoms (mean –4.1; *d*=0.86). Improvements were also observed in work and social functioning (Mean –6.5; *d*=0.73) and resilience (mean 3.9; *d*=0.60). A total of 23% fewer participants endorsed suicidal ideation (PHQ-9 ninth item score >1) at 6 months (16.5%) and 12 months (17.2%) compared to baseline (39.8%).

We observed 12-month mean reductions and respective effect sizes in the severity of depression (mean –6.5; *d*=1.23) and anxiety symptoms (mean –4.3; *d*=0.93). Improvements were also observed in work and social functioning (mean –6.9; *d*=0.76) and resilience (mean 4.3; *d*=0.62). Marked decreases were observed on suicidal ideation (PHQ-9 ninth item score >1) from baseline (39.8%) to 6 months (16.5%) and 12 months (17.2%). Longitudinal trends from baseline for all outcome measures were statistically significant (*P*<.001).

Table 3 presents the effects of program adherence on each clinical measure. The number of lessons completed was significantly associated to lower PHQ-9 (*P*<.001), GAD-7 (*P*<.001), and WSAS scores (*P*<.001) and higher CD-RISC-10 scores (*P*<.001). The number of page views was significantly associated to lower PHQ-9 (*P*<.001), GAD-7 (*P*<.001), and WSAS scores (*P*=.02) and higher CD-RISC-10 scores (*P*<.001) The number of logins was significantly associated only with the PHQ-9 (*P*=.02). No adherence metrics were significantly associated with suicidal ideation.

**Table 3:** Program adherence metric effects on outcome measures.

Adherence metric	PHQ-9^a^		GADS-7^b^		WSAS^c^		CD-RISC-10^d^		PHQ-9, item 9^e^		
	*F* test (*df*)	*P* value (adj)^f^	*F* test (*df*)	*P* value (adj)^f^	*F* test (*df*)	*P* value (adj)^f^	*F* test (*df*)	*P* value (adj)^f^		χ^2^ (*df*)	*P* value (adj)^g^
Logins	7.89 (1)	.02	3.62 (1)	.23	1.00 (1)	>.99	1.09 (1)	>.99		1.18 (1)	>.99
Page views	31.3 (1)	<.001	26.95 (1)	<.001	7.72 (1)	.02	22.81 (1)	<.001		4.83 (1)	.11
Lessons completed	41.42 (1)	<.001	45.65 (1)	<.001	14.72 (1)	<.001	31.69 (1)	<.001		4.57 (1)	.13

^a^Patient Health Questionnaire-9 (PHQ-9) score range = 0 to 27.

^b^Generalized Anxiety Disorder Scale-7 (GAD-7) score range = 0 to 21.

^c^Work and Social Adjustment Scale (WSAS) score range = 0 to 40.

^d^Connor-Davidson Resilience Scale (CD-RISC 10) score range = 0 to 40.

^e^PHQ-9 Item 9, Suicidal Ideation, score range = 0 to 3.

^f^*P* value was associated with the test (*F* test, Type III Tests of Fixed Effects) of the overall time period (week) difference. *P* values adjusted by the Bonferroni method (adj) are shown.

^g^*P* value was associated with the test (χ^2^, Type 3 generalized estimating equations analysis) of the overall time period (week) difference. *P* values are adjusted by the Bonferroni method (adj) are shown.

## Discussion

### Principal Results

This study evaluated the long-term (6- and 12-month) outcomes of a fully automated self-guided (no supportive contacts by email, text, telephone, or face-to-face) video-centric iCBT intervention called Thrive in a rural US community. We believe this is the first study to assess the long-term impacts of an iCBT program within an adult population in rural United States. These analyses focused on participants receiving immediate access to the Thrive intervention. Over the course of 8 weeks, over a third of participants with 8-week data achieved remission, and over half of those maintained remission at 6 months and 12 months. A very low relapse rate was one of the noteworthy observations of this study. In regard to long-term outcomes of primary and secondary measures, study findings demonstrated mean improvements in depression and anxiety symptoms, work and social functioning, and resilience from baseline to 8 weeks. These improvements were sustained at 6 and 12 months. Comparable sustained improvements were also observed with decreased percentages of participants reporting suicidal ideation.

In regard to our adherence analyses, the number of page views and the number of lessons completed most consistently predicted greater sustained positive effects on all outcome measures. Both page views and lessons completed are reasonable markers to assess the program adherence with self-guided iCBT interventions like Thrive, as they indicate the extent to which the users progress through the program. In contrast, the number logins and progress through the program will expectedly vary because some users tend to spend more blocks of time in the program compared to others.

### Comparison With Prior Work

There is limited evidence on the long-term impacts of self-guided iCBT interventions. To our knowledge, only a few studies have examined long-term effects on depression. Mira and colleagues’ study [19] with adults residing in Spain found similar 12-month sustained effects of an iCBT intervention. Clarke and colleagues’ study [20] examined 8-month effects of an iCBT intervention on depression among members of the US Pacific Northwest Kaiser Permanente organization.

Substantial evidence exists regarding CBT’s short- and long-term effectiveness for depression and anxiety [31-33]. A growing number of studies report evidence of equivalent effectiveness of iCBT compared with clinician-delivered CBT [7]. Lorenzo-Luaces and colleagues’ [34] meta-analysis concluded comparable effectiveness of self-guided iCBT with that of antidepressants and in-person psychotherapy. In our trial, Thrive’s 8-week RCT between group (intervention vs control) effect size of 0.63 compared well with “mostly 8-week clinical trials of antidepressants for adults with unipolar major depression” between group (antidepressant vs placebo) effect sizes of 0.27 (Hamilton Depression Rating Scale) and 0.30 (Montgomery-Asberg Depression Rating Scale) in 109 antidepressant medication RCTs [35]. For longer-term outcomes, 12-month relapse rates from remission for Thrive at 8 weeks and STAR*D Phase I citalopram at 12 weeks [36] are 14.2% versus 33.5%, respectively. This comparison includes only Thrive participants with PHQ-9 baseline score 10, consistent with a diagnosis of major depressive disorder.

Adherence in iCBT needs further exploration. In Beintner and colleagues’ review [21], most studies (85%) reported at least 1 adherence indicator, yet adherence metrics varied widely across this literature. The 10 most commonly reported adherence metrics were as follows: full intervention completion; completion of a minimum number of sessions/modules; average number of completed sessions/modules; specified point of discontinuation of the intervention (“dropout”); dropout without specifying a time point; number of participants who were randomized to an intervention group but never logged on; number of times a participant logged on to access the intervention program; total time spent on the program; number of entries into a diary; and number of messages sent to a coach. Recognizing that the specific metric(s) chosen will need to be appropriate to the specific components or delivery formats of online interventions, Beintner and colleagues [21] further recommend the use of multiple adherence metrics, which all studies should report on adherence, providing detailed information on its operationalization.

In our study, we analyzed data on the number of logins, page views, and lessons completed. Page views and lessons completed were the consistent significant predictors of our outcome measures with the exception of suicidal ideation. Cuijpers and colleagues’ [33] meta-analysis of CBT depression studies assessed the number of sessions (comparable to lessons completed in Thrive) as an adherence measure to determine a dose-response effect. In contrast to our findings, they found no significant relationship with study effect sizes. Given the relative infancy of iCBT platforms, it is imperative that future studies critically devise relevant adherence metrics that fit the type of medium.

### Limitations

Our findings need to be considered in light of several limitations. As commonly observed in iCBT studies, assessment completion rates were low with 50.3% and 51.4% of participants completing assessments at 6 and 12 months, respectively. Thus, our results may be skewed due to underlying responder biases. Within-group analyses are limited in that there is no control group with which to compare findings. Relying solely on self-assessments, a common practice in iCBT studies, is a potential weakness of the study; however, the use of validated, widely used instruments largely addressed this issue. The PHQ-9, GAD-7, and WSAS measures correlate well with clinician-administered instruments [24,25,37]; furthermore, they have been shown to be sensitive to treatment effects [26,38,39]. Additionally, self-assessments may underestimate the effect of iCBT compared to clinician-administered assessments [40]. As a community-based trial, our findings cannot be generalized to health care settings. In regard to adherence metrics, we limited our analyses to include the number of logins, page views, and lessons completed. Our study was not originally designed as a dose-response analysis; and therefore, our findings are limited to our post hoc analyses of adherence.

### Conclusions

To our knowledge, this study is the first to demonstrate both short- and long-term positive impacts of a self-guided iCBT intervention on depression and anxiety symptoms, work and social functioning, and resilience among rural adults in the United States. iCBT interventions, such as Thrive, have the potential to provide help and clinical benefit to people with substantial barriers to traditional forms of care; they also present a cost-effective alternative [41] with additional benefits of confidentiality that may not be possible for those seeking traditional care in small rural communities. That iCBT interventions have the potential for greater accessibility, privacy, and affordability in many rural areas compared to in-person psychological treatment is particularly encouraging. Further research is warranted to identify effective and cost-effective dissemination strategies for expanded reach and to understand adoption patterns of iCBT and other internet-based therapies in rural American communities for ensuring optimal uptake of these promising interventions [42].

## Appendix

Multimedia Appendix 1 CONSORT (Consolidated Standards of Reporting Trials) flowchart.

Multimedia Appendix 2 Safety Protocol.

Multimedia Appendix 3 Consort-eHealth.

## Abbreviations

CBT: cognitive behavior therapy
CD-RISC-10: Conner-Davidson Resilience Scale-10
CONSORT: Consolidated Standards of Reporting Trials
GAD-7: Generalized Anxiety Disorder scale-7
iCBT: internet-based cognitive behavior therapy
IRB: institutional review board
PHQ-9: Patient Health Questionnaire-9
RCT: randomized controlled trial
WLC: waitlist control
WSAS: Work and Social Adjustment Scale
