# Correction: Twelve-Month Follow-Up to a Fully Automated Internet-Based Cognitive Behavior Therapy Intervention for Rural Adults With Depression Symptoms: Single-Arm Longitudinal Study

**DOI:** 10.2196/25146

**Published:** 2020-10-23

**Authors:** Mark Schure, Bernadette McCrory, Kathryn Tuchscherer Franklin, John Greist, Ruth Striegel Weissman

**Affiliations:** 1 Department of Health & Human Development Montana State University Bozeman, MT United States; 2 Department of Mechanical & Industrial Engineering Montana State University Bozeman, MT United States; 3 Center for Mental Health Research and Recovery Montana State University Bozeman, MT United States

In “Twelve-Month Follow-Up to a Fully Automated Internet-Based Cognitive Behavior Therapy Intervention for Rural Adults With Depression Symptoms: Single-Arm Longitudinal Study” (J Med Internet Res 2020;22(10):e21336) the authors noted one error.

The affiliation for authors Mark Schure and Kathryn Tuchscherer Franklin was incorrectly listed as:

Department of Mechanical & Industrial Engineering, Montana State University, Bozeman, MT, United States

The correct affiliation for these authors is:

Department of Health & Human Development, Montana State University, Bozeman, MT, United States

This affiliation is affiliation 1 in the corrected manuscript. Accordingly, affiliations 1 and 2 in the originally published manuscript have been renumbered to affiliations 2 and 3 in the corrected manuscript.

The Corresponding Author address for Mark Schure has also been corrected from:

Mark Schure, BSc, MSc, PhD

Department of Mechanical & Industrial Engineering

Montana State University

Bozeman, MT

United States

to:

Mark Schure, BSc, MSc, PhD

Department of Health & Human Development

Montana State University

305 Herrick Hall

Bozeman, MT, 59717

United States

The correction will appear in the online version of the paper on the JMIR Publications website on October 23, 2020, together with the publication of this correction notice. Because this was made after submission to PubMed, PubMed Central, and other full-text repositories, the corrected article has also been resubmitted to those repositories.

