# Supplementary figures and images for: Twelve-Month Follow-Up to a Fully Automated Internet-Based Cognitive Behavior Therapy Intervention for Rural Adults With Depression Symptoms: Single-Arm Longitudinal Study
Source: J Med Internet Res. 2020 Oct 2;22(10):e21336. doi: 10.2196/21336 (PMC7568222; doi:10.2196/21336)

## Multimedia Appendix 1. CONSORT diagram

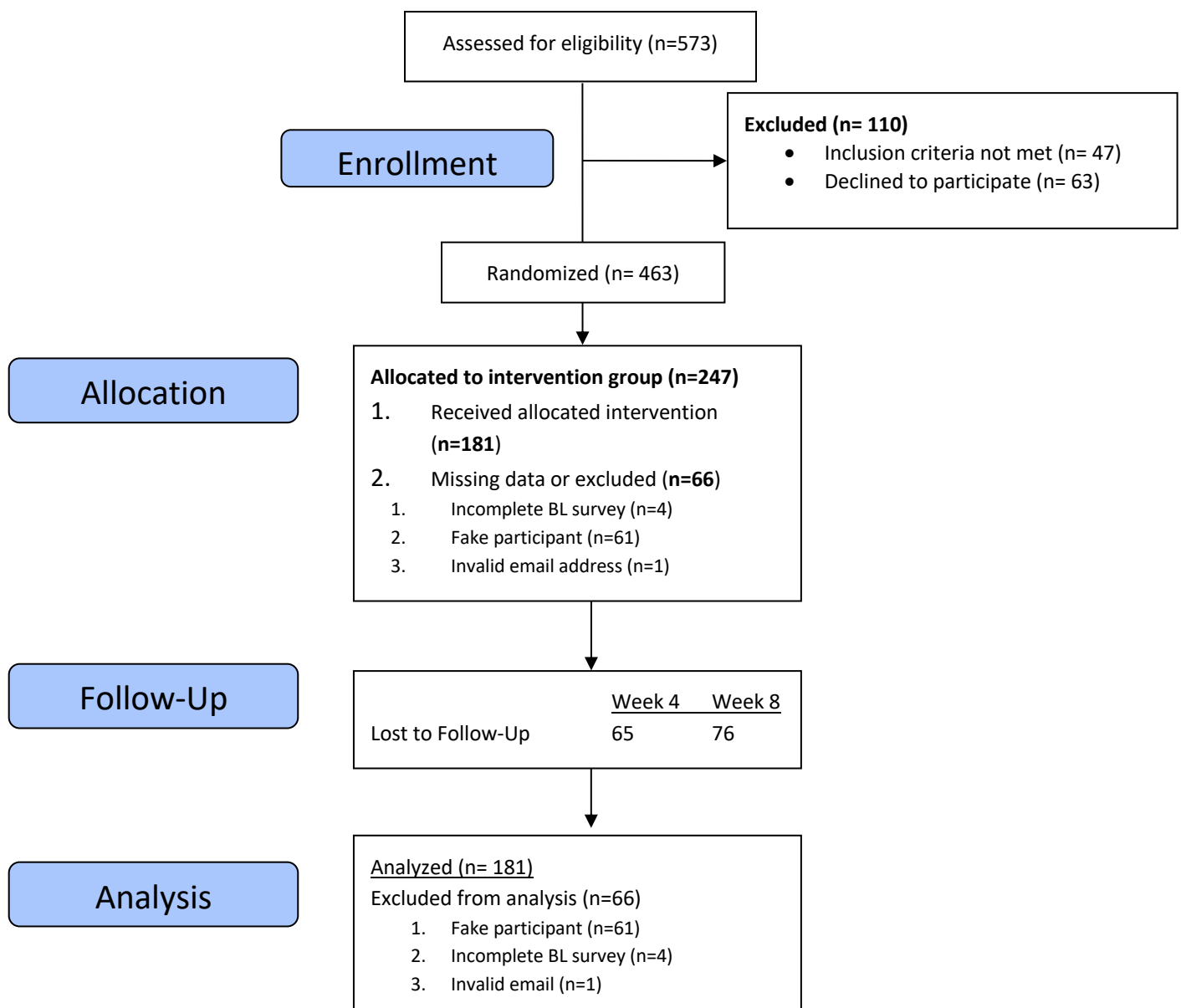

Supplement: Multimedia Appendix 1 [file jmir_v22i10e21336_app1.pdf]
