# Supplementary material for: Twelve-Month Follow-Up to a Fully Automated Internet-Based Cognitive Behavior Therapy Intervention for Rural Adults With Depression Symptoms: Single-Arm Longitudinal Study
Source: J Med Internet Res. 2020 Oct 2;22(10):e21336. doi: 10.2196/21336 (PMC7568222; doi:10.2196/21336)
Supplement: Multimedia Appendix 2 [file jmir_v22i10e21336_app2.pdf]

Multimedia Appendix 2. Safety measures in the study website and Thrive intervention

The study website was the website where study participants applied for and enrolled in the study and completed assessments used as outcome measures. The study website and Thrive contained information designed to increase safety for study participants.

Study Website Information for Potential and Enrolled Participants

The study website home page displayed a link to Montana's National Alliance on Mental Illness resource page.

Study Website Information for Potential Participants with Elevated PHQ-9 Ninth Item Scores at Screening

The nine-item Patient Health Questionnaire (PHQ-9) was administered during enrollment and at each assessment, with item 9 specifically assessing thoughts of death or self-harm. If any potential participant indicated a score ≥0 (indicating at least some recent thoughts of death or self-harm) on item 9 during screening, the study website prompted the individual to seek immediate help by displaying one of the two following messages:

For ineligible individuals:

"Thanks for completing the questionnaire. Based on your answers, you are not eligible to participate in this study. Eligibility requires 1) Montana residency, 2) age 18 or older, 3) having an email address and regular access to a computer, tablet, or smartphone with an Internet connection that's fast enough to watch streaming video and 4) substantially distressed mood. In addition, you indicated that you sometimes think about hurting yourself or think about being dead. If you think you might hurt yourself now, do one of these things right now:

- Call your doctor
- Go to an emergency room
- Call 911
- Call the National Suicide Prevention Lifeline at 1-800-273-TALK (8255)
- Text "Matters" to 741741 to start a text chat with the Crisis Text Line, or
- Visit NowMattersNow.org to learn skills for dealing with thoughts of suicide
- Visit the Montana National Alliance for Mental Illness website at namimt.org/county-resource-guides"

For eligible individuals:

"Thanks for completing the questionnaire. You indicated that you sometimes think about hurting yourself or think about being dead. If you think you might hurt yourself now, do one of these things right now:

- Call your doctor
- Go to an Emergency Room
- Call 911
- Call the National Suicide Prevention Lifeline at 1-800-273-TALK (8255)
- Text "Matters" to 741741 to start a text chat with the Crisis Text Line, or
- Visit NowMattersNow.org to learn skills for dealing with thoughts of suicide
- Visit the Montana National Alliance for Mental Illness website at namimt.org/county-resource-guides

Are you sure you can stay safe? (Individual clicks on one of the two responses,)

[ ] Yes, I'm sure I can stay safe

[ ] No, I'm not sure I can stay safe"

1) **If the individual answers "Yes, I'm sure I can stay safe"**, the study website showed the following text:
   "Thanks for your response. You can continue in the study, but it's important that you seek help if you have frequent or intense thoughts of harming yourself. If you ever feel like you're at risk of hurting yourself, please do one of these things:
   - Call your doctor
   - Go to an emergency room
   - Call 911
   - Call the National Suicide Prevention Lifeline at 1-800-273-TALK (8255)
   - Text "Matters" to 741741 to start a text chat with the Crisis Text Line, or
   - Visit NowMattersNow.org to learn skills for dealing with thoughts of suicide
   - Visit the Montana National Alliance for Mental Illness website at namimt.org/county-resource-guides"

   The participant then is shown the Informed Consent webpage.
2) **If the individual answers "No, I'm not sure I can stay safe**", the study website showed the following text:
   "We're sorry you're not sure you can be safe, but it's good you recognize you're feeling that way. Instead of continuing in this study, please do one of these things right now:
   - Call your doctor
   - Go to an emergency room
   - Call 911
   - Call the National Suicide Prevention Lifeline at 1-800-273-TALK (8255)
   - Text "Matters" to 741741 to start a text chat with the Crisis Text Line, or
   - Visit NowMattersNow.org to learn skills for dealing with thoughts of suicide
   - Visit the Montana National Alliance for Mental Illness website at namimt.org/county-resource-guides"

Study Website Information for Enrolled Participants with Elevated PHQ-9 Ninth Item Scores at Weeks 4 and 8

If an enrolled participant indicated a score ≥0 on item 9 (PHQ-9) at the Week 4 or Week 8 assessment, the study website prompted the individual to seek immediate help by displaying the following message:

> "Thanks for completing the questionnaire. You indicated that you sometimes think about hurting yourself or think about being dead. If you think you might hurt yourself now, do one of these things right now:
> - Call your doctor
> - Go to an emergency room
> - Call 911
> - Call the National Suicide Prevention Lifeline at 1-800-273-TALK (8255)
> - Text "Matters" to 741741 to start a text chat with the Crisis Text Line, or
> - Visit NowMattersNow.org to learn skills for dealing with thoughts of suicide
> - Visit the Montana National Alliance for Mental Illness website at namimt.org/county-resource-guides

If you are sure you can stay safe, please continue to the next page."

Thrive Information for Treatment Group Participants Starting Thrive
Before a participant could start the Thrive curriculum, Thrive presented text, written at the 5th grade reading level, saying that Thrive did not replace the participant's doctor, that instructions provided by Thrive were not provided by a person, and that the participant should seek immediate help if ever in danger of self-harm. Six resources for immediate help (shown in next section) were listed. The participant could not continue in Thrive without checking a box indicating agreement with the information on this page.

Thrive Information for Enrolled Treatment Group Participants with Elevated PHQ-9 Ninth Item Scores
Thrive administered the PHQ-9 at baseline and subsequently attempted repeat assessments approximately every 10 days during participant access to Thrive. If any potential participant indicated a PHQ-9 Item 9 score ≥1, Thrive prompted the individual to seek immediate help by presenting a video reminding the participant that Thrive is not appropriate for individuals who are at risk of hurting themselves and displaying the following message:

"Stop using Thrive if you think you might hurt yourself. Instead, do one of these things right now:

- Call 911 or your doctor
- Go to an emergency room
- Call the National Suicide Prevention Lifeline at 1-800-273-TALK (8255)
- Text 'Matters' to 741741 to start a text chat with the Crisis Text Line, or
- Visit NowMattersNow.org to learn skills for dealing with thoughts of suicide"

Are you sure you can stay safe? (Participant clicks on one of the two responses,)
[ ] Yes, I'm sure I can stay safe
[ ] No, I'm not sure I can stay safe"

1) **If the individual answers "Yes, I'm sure I can stay safe"**, Thrive presented a video suggesting the participant talk with his or her doctor and immediately and displayed text listing the resoures shown above.

2) **If the individual answers "No, I'm not sure I can stay safe**", Thrive presented a video recommending the participant to seek help immediately and displayed text listing the resoures shown above. The participant could not continue in Thrive until indicating that he or she was not in danger of self-harm.

Thrive Information for Enrolled Treatment Group Participants with Elevated PHQ-9 Total Scores
Each time a participant completed the PHQ-9 in Thrive, it presented video feedback to help him/her interpret his/her scores. If the participant's PHQ-9 total score was ≥ 20, the video feedback included a recommendation to talk with a doctor. Additionally, if the participant's PHQ-9 total score was ≥ 10 on the third PHQ-9 completed in Thrive, the video feedback included a recommendation to talk with a doctor.
